# Detecting body dysmorphic disorder in the age of algorithms

**DOI:** 10.3389/frai.2025.1717267

**Published:** 2025-11-25

**Authors:** Diala Haykal, George Kroumpouzos

**Affiliations:** 1 Centre Médical Laser Esthétique Palaiseau, Palaiseau, France; 2 GK Dermatology PC, South Weymouth, MA, United States; 3 Department of Dermatology, Alpert Medical School at Brown University, Providence, RI, United States

**Keywords:** body dysmorphic disorder, body dysmorphia, artificial intelligence, artificial intelligence algorithm, machine learning, aesthetic, aesthetic medicine, aesthetic surgery

## Abstract

Body Dysmorphic Disorder (BDD) is increasingly recognized in the aesthetic practice, yet it remains underdiagnosed and often misunderstood. With its high prevalence, particularly in cosmetic consultations, BDD poses significant ethical and clinical challenges. Aesthetic providers must be vigilant in identifying at-risk individuals and prioritizing psychological well-being alongside procedural outcomes. Artificial Intelligence (AI), with its capacity to analyze behavioral patterns, automate screening tools, and detect subtle indicators of cognitive distortion, presents a new frontier in managing BDD. However, integrating AI into clinical practice requires caution to prevent reinforcing appearance-focused biases and to ensure privacy and fairness. This commentary discusses the opportunities, limitations, and ethical considerations of leveraging AI to assist clinicians in detecting BDD, fostering safer patient outcomes, and advancing the compassionate practice of aesthetic medicine. AI should not accelerate aesthetic procedures but promote reflective, ethically sound decision-making. When integrated responsibly, it can enhance recognition of BDD, support psychological safety, and preserve patient trust through transparency, data protection, and clinician oversight.

## Introduction

Body Dysmorphic Disorder (BDD) is a frequently overlooked yet clinically significant condition in the aesthetic practice (Mataix-Cols et al., 2025). It is characterized by a persistent and intrusive preoccupation with perceived flaws in appearance, typically minor or imperceptible to others. Individuals with BDD often seek repeated cosmetic procedures in hopes of achieving relief, but they typically find little psychological benefit from such interventions (Laughter et al., 2023). In aesthetic settings, where consultations are often brief and focused on procedural planning, the disorder may go unrecognized, especially when masked by articulate aesthetic goals and a desire for perfection. This diagnostic gap carries ethical risks, as well-intended interventions may inadvertently reinforce distorted self-perceptions. As digital technologies become more integrated into aesthetic practice, interest is growing in how artificial intelligence (AI) might assist clinicians in identifying early BDD signs. By analyzing behavioral patterns, linguistic expressions, and visual cues, AI may provide a new layer of insight to support clinical judgment, without replacing it (Türk et al., 2023; Türk et al., 2023).

This commentary explores the potential role of AI in improving the recognition and management of BDD within aesthetic dermatology. It addresses both the promise and limitations of using AI in this context. We advocate for a care model that utilizes technology not to expedite procedures, but rather to foster greater psychological awareness, ethical restraint, and patient-centered decision-making. In this context, AI tools are not intended to replace clinicians or mental health professionals. Rather, they can be deployed before or during aesthetic consultations by trained providers as preliminary decision-support systems, flagging patients who may require further psychological evaluation instead of cosmetic procedures.

### The hidden burden of BDD in aesthetic practice

BDD remains an often overlooked yet profoundly impactful condition in aesthetic and reconstructive settings. While surgeons and cosmetic practitioners are skilled in assessing anatomical harmony and procedural indications, they may be less attuned to discerning when a patient’s concerns stem from psychological distress rather than genuine morphological abnormalities. BDD is characterized by significant emotional suffering and impaired social or occupational functioning. Recognizing and addressing BDD is essential, as untreated cases can lead to poor satisfaction, repeated procedures, and worsening mental health outcomes (Toh et al., 2025).

A study comparing dermatologic patients found that 14.0% of individuals seeking cosmetic procedures met the diagnostic criteria for BDD. This rate is significantly higher rate than the 6.7% found in general dermatology patients and just 2% in control groups (Kaleeny and Janis, 2024). In contrast, among broader dermatologic patients (non-cosmetic intent) the prevalence has been estimated at approximately 12.5%, while estimates in the general population remain around 1–3% (Saade et al., 2024).

### The role of AI in identifying BDD

BDD recognition includes detailed history and in-office observation of the patient, interview, and ‘pen and pencil’ tools. Red flags from the interview, history, and observations, such as being unsatisfied with all previous procedures or history or current symptoms of psychiatric disorder, are extremely valuable in establishing a BDD diagnosis (Toms et al., 2025). AI offers a novel opportunity to enhance the recognition of BDD in aesthetic settings. Although not designed to diagnose psychiatric disorders, AI systems can analyze behavioral, linguistic, and visual patterns that may signal underlying dysmorphia. These technologies include digital phenotyping, image analysis, and natural language processing tools that provide contextual insights that go beyond what is perceptible in standard clinical encounters (Landau et al., 2025).

In this context, AI systems can help distinguish BDD from normal appearance concerns that are related to a lesser degree of appearance dissatisfaction. A normal appearance dissatisfaction is often situational, proportionate, and transient, such as mild concern before a major life event or desire for subtle aesthetic improvement. Individuals with non-pathological concerns generally exhibit realistic expectations and a balanced perception of their features, whereas those with BDD fixate on minor or imagined flaws, experience significant emotional distress, and may pursue multiple cosmetic procedures despite having little to no observable defects. By identifying these subtleties, AI can support clinicians in distinguishing between adaptive aesthetic motivation and potential psychopathology, ultimately promoting safer and more ethical decision-making in cosmetic practices (Sejdiu et al., 2024).

Digital phenotyping analyzes smartphone-based behaviors, such as frequent use of filters, facial editing apps, or repeated photo comparisons, all of which are often linked to self-image dysregulation. Image-based algorithms can evaluate the objective symmetry or proportion of a patient’s face in contrast to their reported dissatisfaction, highlighting disproportionate concerns. Additionally, natural language models embedded in digital forms or chatbots can detect repetitive language, intense emotional tone, negative self-referencing, and other cues associated with dysmorphic thinking (Tan et al., 2024; Costilla-Reyes and Talbot, 2025). AI systems are not designed to diagnose psychiatric disorders or make treatment decisions autonomously. Instead, they are intended to serve surgeons, dermatologists, and mental health professionals as early alert systems, particularly during pre-procedure digital intake or first consultations, to prompt more thorough psychological assessment when needed.

Importantly, these tools serve as cognitive aids for clinicians by flagging patterns that warrant deeper exploration. AI-generated alerts may prompt the physician to slow the consultation, thoroughly investigate the patient’s history and motivations, and initiate essential discussions about the need for psychological evaluation. In this manner, AI functions not merely as a replacement for clinical judgment but as a reflective checkpoint in fast-paced aesthetic practice.

### Ethical imperatives and cautions

The integration of AI into clinical decision-making raises important ethical considerations. AI models are only as good as the data on which they are trained. If built on filtered, idealized images or culturally narrow beauty standards, they risk perpetuating harmful biases and promoting unrealistic expectations (Haykal et al., 2023; Haykal and Cartier, 2024; Haykal et al., 2024). There is a danger that AI could inadvertently validate patients’ distorted self-perceptions, especially if the algorithm’s definition of “aesthetic improvement” aligns with artificial norms (Daneshjou et al., 2021).

Privacy and consent also become crucial issues. Patients must be explicitly informed when their behavior, language, or digital data are being analyzed. Informed consent should extend beyond the procedure to encompass the technological tools used in evaluation. Furthermore, the psychological implications of being “flagged” by an AI system must be handled with empathy and professionalism to prevent stigmatization and preserve trust (Meadi et al., 2025).

Clinicians must assert control over final decisions and be prepared to contextualize or disregard AI outputs when human judgment suggests a more suitable course. The objective is not to deny care, but to guide patients toward the form of care they most need, which may involve psychological support rather than merely procedural solutions. For AI to function reliably, it must analyze patient-derived linguistic, behavioral, or image-based data. Therefore, no AI assessment should occur without prior informed consent. Patients must agree to digital analysis before their data is captured, with the option to decline without affecting their access to care. When consent is denied, clinicians rely exclusively on traditional, non-digital assessments (Singh et al., 2024; Kenig et al., 2024).

### Informed consent and data protection

Before any behavioral, linguistic, or image-based data are analyzed using AI systems, patients must be informed and provide explicit consent. This digital consent must be separate from procedural consent and should clarify the purpose, type of data collected, storage duration, and the right to withdraw. Data should be anonymized, encrypted, and stored within secure clinical infrastructures that prohibit sharing with insurance companies, commercial platforms, or non-medical third parties. If a patient declines data analysis, traditional clinician-led assessment must remain fully available with no impact on access to care. To prevent misuse, AI-derived data must remain within secure, encrypted medical infrastructures and never be shared with third parties such as insurance companies, commercial platforms, or employers. Access should be limited to the clinical team involved in patient care, with audit trails and ethical oversight to prevent secondary use or commercialization of sensitive data.

### Clinical integration and training implications

The effective use of AI in BDD screening is achieved through thoughtful and unobtrusive integration into existing workflows. Digital intake forms or virtual consultations can include subtle behavioral assessments or validated screening questionnaires powered by AI. These tools can triage patients before they reach the consultation room identifying red flags for BDD based on the patient’s history. This equips practitioners with early insights into the emotional and cognitive context of the request (Pereira et al., 2023). Additionally, AI can contribute to provider education. By simulating real-world patient interactions and flagging psychological red flags, AI-driven training programs can teach clinicians to recognize patterns of dysmorphic concern. This training is valuable, especially in environments where mental health education is not systematically included in aesthetic curricula. Additionally, in individuals with an established BDD diagnosis, interpretable machine learning systems may predict the response to treatment, paving the way for more targeted, personalized, and ultimately efficacious interventions (Landau et al., 2025).

By leveraging AI to deepen our diagnostic awareness, we can foster a more psychologically informed approach to aesthetic medicine–one that sees beauty as more than skin deep and success as extending beyond mere technical perfection.

### Seeing patients beyond the surface

The cosmetic consultation process is increasingly shaped by technology. Patients often arrive influenced by algorithmically curated beauty standards, filters, and social comparison through digital platforms (Haykal, 2022). If the aesthetic community fails to critically engage with this digital landscape, it risks becoming complicit in a system that reinforces distress rather than fostering healing (Walker et al., 2021).

When applied ethically, AI can provide more than mere precision and efficiency; it provokes essential reflection. It allows us to slow down in a field that often hastily moves from request to intervention. Most importantly, AI can help us see patients more clearly, not just as aesthetic projects, but as individuals seeking validation, relief, and wholeness.

The most advanced technology in aesthetic medicine is not necessarily the devices that lift or tighten. Instead, it may be the tools that gently and wisely advises us when not to proceed with treatment.

## Conclusion

AI is not a panacea for the challenges posed by BDD, but it serves as a valuable tool for early recognition and ethical decision-making in aesthetic practice. As aesthetic practitioners, we are entrusted with the responsibility not only to enhance our patients’ appearances but also to safeguard their psychological well-being. For individuals with BDD, aesthetic interventions can increase distress if the underlying disorder goes unrecognized. AI has the potential to prompt reflection, highlight concerning patterns, and enhance our clinical sensitivity, provided it is implemented with transparency, empathy, and restraint. Rather than hastening aesthetic care, AI should help us slow down, ask better questions, and engage with our patients more comprehensively. This approach can shift the practice of aesthetic medicine toward a model that values insight as much as outcomes and prioritizes care alongside correction.

## Data Availability

The original contributions presented in the study are included in the article/supplementary material, further inquiries can be directed to the corresponding author/s.
